# Supplementary material for: Female Human Papillomavirus Infection Associated with Increased Risk of Infertility: A Nationwide Population-Based Cohort Study
Source: Int J Environ Res Public Health. 2020 Sep 7;17(18):6505. doi: 10.3390/ijerph17186505 (PMC7558487; doi:10.3390/ijerph17186505)
Supplement: Supplementary file 1 [file ijerph-17-06505-s001.pdf]

Supplementary Table 1. Association of HPV and Infertility with different variables and subgroup analyses.

	non-HPV			HPV							
**Variables**	**n**	**PY**	**IR**	**n**	**PY**	**IR**	**cHR**	**(95% CI)**	**aHR†**	**(95% CI)**	***p*-value for interaction**
Age, years											0.176
15–25	105	26872	3.91	121	26816	4.51	1.16	(0.89,1.51)	1.15	(0.89,1.5)	
26–35	148	20211	7.32	227	20145	11.3	1.54	(1.25,1.89)***	**1.53**	(1.24,1.88)***	
36–45	20	17886	1.12	35	18072	1.94	1.74	(1.01,3.02)*	1.73	(1.00,3.00)	
Comorbidities											
endometriosis											0.322
No	252	62865	4.01	359	62745	5.72	1.43	(1.22,1.68)***	1.43	(1.22,1.68)***	
Yes	21	2104	9.98	24	2287	10.5	1.06	(0.59,1.91)	1.03	(0.57,1.86)	
PCOS											0.086
No	258	64118	4.02	371	64102	5.79	1.44	(1.23,1.69)***	1.44	(1.22,1.68)***	
Yes	15	852	17.6	12	930	12.9	0.73	(0.34,1.56)	0.75	(0.35,1.61)	
Benign neoplasm of ovary											0.494
No	247	62152	3.97	351	62086	5.65	1.42	(1.21,1.68)***	1.42	(1.21,1.67)***	
Yes	26	2817	9.23	32	2946	10.9	1.18	(0.71,1.99)	1.16	(0.69,1.95)	
PID											0.584
No	265	64031	4.14	373	63954	5.83	1.41	(1.21,1.65)***	1.41	(1.2,1.65)***	
Yes	8	939	8.52	10	1079	9.27	1.09	(0.43,2.77)	1.05	(0.41,2.68)	
Uterine leiomyoma											0.337
No	259	62589	4.14	368	62447	5.89	1.43	(1.22,1.67)***	1.42	(1.21,1.66)***	
Yes	14	2380	5.88	15	2585	5.8	1	(0.48,2.06)	1.02	(0.49,2.12)	
Infertility-associated operation											0.511
No	264	62898	4.2	373	62766	5.94	1.42	(1.21,1.66)***	1.41	(1.2,1.65)***	
Yes	9	2072	4.34	10	2266	4.41	1.03	(0.42,2.54)	1.15	(0.47,2.84)	

*: *p*-value<0.05;-***: *p*-value<0.001;

PCOS: polycystic ovarian syndrome; PID: pelvic inflammatory disease;

n: number of patients ;PY: person-year; IR: incidence rate per 1000 person-years; cHR: crude hazard ratio; aHR: adjusted hazard ratio;

†: multivariable model with HPV, age, endometriosis, PCOS, benign neoplasm of ovary and PID.
